# Andrographolide promote the growth and immunity of *Litopenaeus vannamei*, and protects shrimps against *Vibrio alginolyticus* by regulating inflammation and apoptosis *via* a ROS-JNK dependent pathway

**DOI:** 10.3389/fimmu.2022.990297

**Published:** 2022-09-09

**Authors:** Xiaoli Yin, Xueqi Zhuang, Weitao Luo, Meiqiu Liao, Lin Huang, Qiqian Cui, Jiayi Huang, Chunxia Yan, Zixiang Jiang, Yuan Liu, Weina Wang

**Affiliations:** Guangzhou Key Laboratory of Subtropical Biodiversity and Biomonitoring, Guangdong Provincial Key Laboratory for Healthy and Safe Aquaculture, Key Laboratory of Ecology and Environmental Science in Guangdong Higher Education, College of Life Science, South China Normal University, Guangzhou, China

**Keywords:** andrographolide, *Litopenaeus vannamei*, *Vibrio alginolyticus*, ROS, JNK, inflammation, apoptosis

## Abstract

*Vibrio alginolyticus* (*V. alginolyticus*) is one of the major pathogens causing mass mortality of shrimps worldwide, affecting energy metabolism, immune response and development of shrimps. In the context of the prohibition of antibiotics, it is necessary to develop a drug that can protect shrimp from *V. alginolyticus*. Andrographolide (hereinafter called Andr), a traditional drug used in Chinese medicine, which possesses diverse biological effects including anti-bacteria, antioxidant, immune regulation. In this study, we investigated the effect of Andr on growth, immunity, and resistance to *V. alginolyticus* infection of *Litopenaeus vannamei* (*L. vannamei*) and elucidate the underlying molecular mechanisms. Four diets were formulated by adding Andr at the dosage of 0 g/kg (Control), 0.5 g/kg, 1 g/kg, and 2 g/kg in the basal diet, respectively. Each diet was randomly fed to one group with three replicates of shrimps in a 4-week feeding trial. The results showed that dietary Andr improved the growth performance and non-specific immune function of shrimps. *L. vannamei* fed with Andr diets showed lower mortality after being challenged by *V. alginolyticus*. After 6 h of *V. alginolyticus* infection, reactive oxygen species (ROS) production, tissue injury, apoptosis, expression of inflammatory factors (IL-1 β and TNFα) and apoptosis-related genes (Bax, caspase3 and p53) were increased in hemocytes and hepatopancreas, while feeding diet with 0.5 g/kg Andr could inhibit the increase. Considering that JNK are important mediators of apoptosis, we examined the influence of Andr on JNK activity during *V. alginolyticus* infection. We found that Andr inhibited JNK activation induced by *V. alginolyticus* infection on *L. vannamei.* The ROS scavenger N-acetyl-l-cysteine (NAC) suppressed *V. alginolyticus*-induced inflammation and apoptosis, suggesting that ROS play an important role in *V. alginolyticus*-induced inflammation and apoptosis. Treated cells with JNK specific activator anisomycin, the inflammation and apoptosis inhibited by Andr were counteracted. Collectively, Andr promote the growth and immunity of *L. vannamei*, and protects shrimps against *V. alginolyticus* by regulating inflammation and apoptosis *via* a ROS-JNK dependent pathway. These results improve the understanding of the pathogenesis of *V. alginolyticus* infection and provide clues to the development of effective drugs against *V. alginolyticus*.

## Introduction

*Vibrio alginolyticus* (*V. alginolyticus*), a Gram-negative marine *Vibrio*, is ubiquitously found in the ocean and offshore coastal and estuarine areas. According to an increasing number of studies, *V. alginolyticus* is not only limited to infecting marine species such as oysters (1), groupers (2), and shrimps (3), but also gradually become an opportunistic pathogen of human infection (4–6). Over the last decade, aquatic animal diseases caused by *V. alginolyticus* occur frequently with the expansion of aquaculture scale and the deterioration of water environment, leading to enormous economic losses to the aquaculture industry (7–10). In recent years, research on the pathogenic mechanisms of *V. alginolyticus* has gained momentum. A number of key virulence factors including the iron uptake system, lipopolysaccharide (LPS), haemolysin, extracellular proteases and type III secretion system (T3SS), likely play a role in its pathogenesis (11–17).

Invertebrates such as shrimp and crab, due to lack of adaptive immunity unique to vertebrates, mainly rely on innate immune system to resist the invasion of bacteria and viruses (18). The innate immune system possesses many arsenals to fight off pathogens, include antimicrobial peptides at mucosal surfaces, activation of the complement system in the blood. Chemoattraction of immune cells to the infection site and pattern recognition receptors that evolved to sense pathogen-associated molecular patterns and to consequently activate inflammatory pathways required for pathogen elimination (19). In addition, phagocytic immune cells, such as neutrophils and macrophages, are important effector cells mediating inflammatory response and reactive oxygen species (ROS) production for host defense, and play an important role in nonspecific immune defense. Under normal physiological conditions, neutrophils proliferation and apoptosis maintain a dynamic balance, which is conducive to both the body’s defense response and the dissipation of inflammatory response. However, apoptosis can be accelerated under the action of many factors such as LPS and TNFα. Within the host, physiological clearance systems such as superoxide dismutase (SOD) and catalase mediate ROS levels and prevent damage to host molecules. If homeostasis is disrupted, uncontrolled ROS production will result in oxidative stress, excessive inflammatory responses, tissue damage, apoptosis (20) and necrosis, thus detrimental to host defense (21). Our previous study found that the ROS production and DNA damage accumulation in hemocytes increased after *V. alginolyticus* infected *L. vannamei* for 6 h, leading to a decrease in THC (22). In addition, studies have found that ROS formation was accelerated during *V. alginolyticus* ^WT^ infection, promoting hemocyte apoptosis in the oyster and fish (14, 23). VscC, VopS and VopQ in *V. alginolyticus* T3SS is involved in the induction of apoptosis (14, 23). Therefore, controlling ROS production, inflammation and inhibiting apoptosis can effectively reduce the damage caused by bacterial infection (24). In aquaculture, many studies reported that adding natural substances with anti-inflammatory effect to feed could improve the survival rate of aquatic animals under bacterial infection stress (25, 26). On the one hand, adding natural substances in the feed can improve the non-specific immune function of aquatic animals (27); on the other hand, natural substances can control the inflammatory response of the body and avoid excessive inflammatory response to the body damage in the process of bacterial infection (28). In recent years, Chinese herbal feed additives are more and more favored by people with the implementation of the policy of banning antibiotics in China.

Andrographolide (Andr), a labdane diterpenoid and the major constituent of *A. paniculata*, has been widely used in Asia as herbal medicine (29, 30). Andr possesses diverse biological effects including anti-viral (31), anti-bacteria (32), antioxidant, immune regulation, gastrointestinal protective effects, hepatoprotective (33), anti-cancer (34) and anti-inflammatory (35) properties. Clinical studies have demonstrated that Andr could be useful in therapy for a wide range of diseases such as osteoarthritis, upper respiratory diseases, multiple sclerosis, fever, hepatic and neural toxicity, cancer, etc. (36, 37). As a natural anti-inflammatory drug, Andr reduces the expression of several proinflammatory genes, including cyclooxygenase-2 (COX-2), IL-6, IL-8, IL-1β, TNFα and inducible nitric oxide synthase (iNOS) (38, 39). The proinflammatory and hemodynamic effects of lipopolysaccharide (LPS) were decreased by intragastric or intraperitoneal injection of 1 mg/kg Andr (40, 41). In addition, Andr also protects cells from oxidative stress (42) and can inhibit TNF-α-induced ROS generation, Src phosphorylation, NADPH oxidase activation, and ICAM-1 expression (43, 44). Previous study have shown that Andr can target multiple inflammatory cytokines related pathways, such as NF- κB, AP-1, HIF-1, PI3K/Akt, MAPK, JNK, JAK/STAT and so on (45). Furthermore, Andr mainly initiates immune responses by regulating complementary systems of the body, granulocytes and macrophages, which play a very important role in overcoming various diseases and infections in immunodeficient patients (46, 47). The purified diterpene Andr has been reported to stimulate non-specific immune responses in mice (48) and *Labeo rohita* (49). Although Andr has been reported as a feed additive in aquatic animals (49, 50), few studies have explored the specific mechanisms of Andr on bacteria-induced inflammation and apoptosis. In the present study, different levels of Andr were supplemented into basal diets to investigate the effects of Andr on growth performance, immunity, resistance to *V. alginolyticus* and its mechanism of *L. vannamei*. The results could contribute to the healthy cultivation of *L. vannamei*.

## Materials and methods

### Preparation of experimental diet

The Andr (HPLC≥98%) was procured from Yuanye Biotechnology Co., Ltd. (Shanghai, China). Four experimental diets were basal diet (Control), basal diet supplemented with 0.5, 1 and 2 g/kg Andr. Basal diet was purchased from Shuyuan Aquatic Science and Technology Co., LTD (Guangdong, China). Pulverize the basal diet with a grinder and sieve it using a nylon sieve (300 mesh size). The experimental diets were prepared by mixing the basal diet pulverized powder and different concentrate of Andr, then adding 40% water until a stiff dough was obtained. The dough was pelletized by double-screwextruders (F-26 [III], China) with diameters of 1.0 mm. The difference of Andr supplemental amount in each group was replaced by the same amount of basal feed pulverized powder.

### Experimental design

Healthy shrimps (*L. vannamei*) free of bacterial and viral infections were collected from an aquaculture farm in Panyu, Guangdong Province, China. They were acclimatized in fiberglass tanks with 10 ± 1 ppt seawater in an aeration system for 7 days. During acclimatization, the shrimps were fed 3 times (08:00, 14:00 and 20:00) per day with commercial feed (Shuyuan Aquatic Science and Technology Co., LTD, Guangdong, China). Shrimps in the intermolt stage (stage C) were selected for the experiments. A total of 240 tails *L. vannamei* shrimps (weight of 2.311 ± 0.105 g) were randomly distributed into twelve 100 L glass aquaria containing 50 L of sea water (3 replicates of 4 treatments, randomly assigned 20 tails/aquarium). Experimental diets were fed to shrimp in each group 3 times (08:00, 14:00 and 20:00) daily for 4 weeks. The daily feeding quantity was 5%–8% of body weight and adjusted according to previous feeding response. During the experiment, the water temperature was maintained at 28 ± 2°C, salinity at 10 ± 1 ppt, and pH at 8.2 ± 0.2.

### Investigation of growth performance

After the 4-week feeding trial, all shrimps were fasted for 24 h and then weighed to calculate weight gain rate (WG), specific growth rate (SGR), feed conversion ratio (FCR) and survival rate (SR) according to the following equations (51):


Weight gain rate (WG) = [(Final weight − Initial weight) / Initial weight] ×100;

 Specific growth rate (SGR) = (ln Final weight − ln Initial weight / Experimental days) × 100;

Feed conversion ratio (FCR) = Feed intake (FI) / (Final weight (g) – Initial weight (g));

Survival rate (SR)=Number of final shrimps/Number of initial shrimps × 100;

### Challenge test

The *V. alginolyticus* was cultured overnight at 37°C using Luria-Bertani (LB) medium. Then, bacteria solution was harvested and washed three times with sterilized phosphate-buffered saline (PBS) by centrifugation at 3000 rpm for 10 min. After the 4-week feeding regime, the bacterial challenge test was carried out. Shrimps were fasted 24 h before trial to avoid interference of diet. Forty shrimps from each treatment were challenged with *V. alginolyticus* by injecting 10 μL of bacterial suspension into the ventral sinus of the shrimp at a dose of 10^7^ colony forming units (CFU) ml^-1^. While, the shrimps that received no Andr and received *V. alginolyticus* suspension (10 μL) or only PBS (10 μL) performed as the challenged control and unchallenged control, respectively. Experimental shrimps were kept into ten plastic aquariums (50 × 38 × 30 cm, randomly assigned 20 tails/aquarium). Each aquarium was provided with continuous aeration during the challenge test. The mortality in each replicate tank was recorded for 7 days. The SR was calculated using the following formula:


SR(%)=(number of shrimps that survived)/(number of shrimps that injected)×100

### Primary hemocyte culture of shrimp

Shrimp hemocytes were filtered out with a 70 mm filter membrane. Then the hemocytes were washed with PBS twice. Leibovitz’s−15 (L-15) (ThermoFisher, MA, US) medium containing 0.1% penicillin-streptomycin solution and 10% fetal bovine serum (FBS) (ThermoFisher, MA, US) was used to resuspend the hemocytes. The hemocyte seeded into 24 well plates at the density of 1 × 10^6^/well and cultured in a CO_2_ free incubator at 28°C. Trypan blue staining was used to determine the cell viability of primary hemocytes. If the positive rate of trypan blue staining of primary hemocyte was less than 5%, cells isolated from that shrimp could be used for further experiments. The treatment experiment can be carried out after the hemocytes were cultured to semi-adherent. Except the control group, the other groups were treated with inactivated *V. alginolyticus* for 3 h. Then, the corresponding concentration of Andr, N-acetyl-L-cysteine (NAC) or anisomycin was added to inhibit the ROS generation or activate the JNK signaling of hemocytes respectively.

### Phagocytic activity assay

Three shrimps of each group were used to investigate the phagocytic activity of hemocyte in *L. vannamei*. Hemocytes were adjusted to 1 × 10^7^ cells/mL using Leibovitz’s L-15 medium supplemented with 10% FBS. Hemocytes were incubated with 0.5 µm microspheres (Red beads) (Huge biotechnology, Shanghai, China) at a 1:20 (cells: beads) ratio for 4 h at 28°C, respectively. After incubation, the cells were collected and centrifuged at 100 × g for 10 min at 4°C to remove excess beads. Then the cells were resuspended in 1 mL PBS. After three times washes with PBS, the phagocytic activities of hemocyte from each group were independently analyzed by using BD Arial III flowcytometer (BD, USA). Hemocytes incubated without microspheres were also included as blank controls. Data analyses were performed using FlowJo X. Phagocytic activities of hemocytes were expressed as phagocytic rate and phagocytic index, were calculated as:


Phagocytic rate (%) = (Phagocytic hemocytes)/ (Total hemocytes) × 100

Phagocytic index=(Beads per phagocytic cell)/(Number of phagocytic cells)

### Total RNA isolation and quantitative real-time PCR

The total RNA in each sample was extracted using Trizol (Vazyme Biotech, Nanjing, China) following the manufacturer’s instructions. The RNA concentration and quality were assessed with NanoDrop ND-2000 Spectrophotometer (Thermo Scientific, Waltham, MA, USA). The integrity of extracted RNA was determined by electrophoresis on a 1.2%(w/v) agarose gel. After that, 1 μg total RNA was reversely transcribed to cDNA in 20 μl reactions using HiScript III 1st Strand cDNA Synthesis Kit (+gDNA wiper) (R312-01, Vazyme, Nanjing, China) according to the manufacturer’s instructions.

Then, qRT-PCR was performed in a total 20 μl volume: 2 μl of cDNA template; 0.5 μl of Forward primer (10 mM); 0.5 μl of Reverse primer (10 mM); 6.6 μl of Nuclease-free treated water (TransGen Biotech, Beijing, China); 0.4 μl of Passive Reference Dye II (TransGen Biotech, Beijing, China) and 10 μl of TransStart^®^ Top Green qPCR SuperMix (AQ131-01, TransGen Biotech, Beijing, China). A two-step real-time qPCR was carried out in an ABI 7500 Thermal Cycler (Applied Biosystems, Foster City, CA) with amplification program as follows: 95°C for 10 min and then 40 cycles of 95°C for 15 s and 60°C for 60 s. All primers used in RT-qPCR are shown in **Table S1**. The relative values of gene expression levels were expressed as 2^−ΔΔCt^. All experiments were repeated three times.

### Antioxidant enzyme detection

Collect the blood with an anticoagulant tube and mix it upside down. Centrifuge at 600 g at 4°C for 10 min, remove the supernatant into a new 1 mL centrifuge tube. After being diluted twice with PBS, it can be used as a plasma sample for testing. The hepatopancreas tissues of shrimps were accurately weighed, and 9 times the volume of PBS was added according to 1:9. Mechanical homogenization was performed under ice water bath, and centrifugation was conducted for 10 min at 2500 rpm. Supernatant was taken for testing. Superoxide dismutase (SOD), catalase (CAT), total antioxidant capacity (T-AOC) and malondialdehyde (MDA) were detected using relevant assay kits (Beyotime Biotechnology, Shanghai, China) following the manufacturer’s instructions.

### Histopathology

The hepatopancreas tissue of *L. vannamei* were immobilized in 10% formalin for 24 h and then dehydrated in increments of alcohol (50%–95%). The dehydrated tissues were embedded in paraffin and cut into 4 μm thick slices. Tissues sections were stained with hematoxylin and eosin (H&E), then examined using a light microscope (DM6, Leica, Germany).

### Determination of intracellular ROS generation

To measure the production of ROS by hemocytes, the cell-permeant probe 2’,7’-dichlorofluorescein diacetate (DCFH-DA) kit (Beyotime, Shanghai, China) was used according to the manufacturer’s instructions. The hemolymph and anticoagulant mixture were incubated in the dark for 30 min with 10 μM DCFH-DA. The cell suspension was prepared after washing with PBS for 3 times. Then, the fluorescence of the cell suspensions was recorded using flow cytometry. ROS production is represented by the mean DCF fluorescence. For each sample, 10,000 events were collected.

### Apoptosis assay

Hemocytes of shrimp were collected and resuspended with PBS. Subsequently, cells were mixed with binding buffer 100 μL. The apoptosis levels of cells were detected with Annexin V-FITC/PI apoptosis detection Kit (Yeasen Biotechnology, Shanghai, China) according to manufacturer’s protocol. Annexin V-FITC 5 L and PI 10 μL were added into the mixture and incubated for 10 min at room temperature in the dark. The apoptosis levels were analyzed under flowjo_V10 software.

### TUNEL assay

Apoptosis was determined by the TUNEL detection apoptosis kit (Elabscience, Wuhan, China). The sections of hepatopancreas tissue were deparaffinized in 1X Dewaxing solution for 50 min at 60°C and then incubated with 1X proteinase K for 20 min at 37°C. After washing three times with PBS buffer, the sections were incubated in TdT equilibration buffer for 30 min, then incubated with 50 μL TUNEL reaction mixture containing 35 μL of TdT equilibration buffer, 10 μL of AF488-labeling solution and 5 μL of TdT Enzyme for 60 min at 37°C in the dark. Then, the slides were rinsed in PBS three times and nuclei were counterstained with 4, 6-diamidino-2-phenylindole dihydrochloride (DAPI) for 5 min at room temperature in the dark. The specimen was covered in streptavidin‐HRP for 30 min and washed in PBS three times. The slides were rinsed in PBS four times and observed by microscope (DM6, Leica, Germany).

Hemocytes were treated with *V. alginolyticus* or Andr or NAC or Anisomycin and then cultured 24 h. After rinsing three times with PBS, the cells were fixed with 4% paraformaldehyde for 20 min and permeabilized in 0.2% Triton-X 100 for 30 min at room temperature. The labeling process of cell slides were the same as hepatopancreas tissue sections. The results of cell slide were photographed using the laser confocal scanning microscopy (Carl Zeiss LSM 800). TUNEL‐positive cells were counted, and the apoptotic index was calculated as a ratio of (apoptotic cell number)/(total cell number) in each field. All experiments were conducted in triplicate.

### Western blotting analysis

After extracting the proteins from the hepatopancreas samples collected from shrimps of each group were lysed in the RIPA lysis buffer (Beyotime, China) for 30 min on the ice. The sample lysates were centrifuged (12,000 g, 10 min) at 4°C, and the supernatant was transferred to a new sterile tube and store to -20°C. Total protein was quantified using BCA Protein Quantification Kit (Yeasen, Shanghai, China). The protein samples were isolated by 12% sodium dodecyl sulfate-polyacrylamide gel electrophoresis (SDS-PAGE), and transferred to polyvinylidene difluoride (PVDF) membranes (GE healthcare, Uppsala, Sweden). The membranes were blocked with 5% nonfat dried milk in TBST (containing 0.05% Tween-20) and incubated with primary antibodies (JNK1/2/3 rabbit monoclonal antibody, Phospho-JNK1/2/3 rabbit monoclonal antibody, β-Actin mouse monoclonal antibody) overnight at 4°C. Bound antibodies were visualized with AP-labeled goat anti-rabbit IgG (H+L) or AP-labeled goat anti- mouse IgG (H+L) and immunoreactivity assessed by chemiluminescence reaction, using the NBT/BCIP western detection system. Densitometric scanning analysis was performed using NIH ImageJ 7 software.

### Statistical analysis

All analyses were implemented in GraphPad Prism V8.0.2 (GraphPad, San Diego, CA, USA). And the differences between the different groups were analyzed by one-way analysis of variance (ANOVA) using SPSS software version 18.0 (IBM, Armonk, NY, USA). All of the experiments described in this study were conducted in triplicates, and data was presented as the mean ± standard error. *p<* 0.05 was considered statistically significant.

## Results

### The effect of Andr supplementation on growth performance of *L. vannamei*

**Figure 1:**
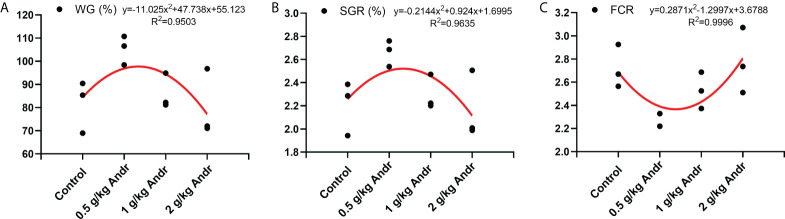
The relationship between WG (%) **(A)**, SGR (%) **(B)**, FCR **(C)** of *L. vannamei* and different levels of Andr. WG, weight gain rate; SGR, specific growth rate; FCR, Feed conversion ratio.


*L. vannamei* fed diets with 0.5 g/kg Andr showed significantly higher WG and SGR than the shrimps fed diet with 0 g/kg, 1 g/kg and 2 g/kg Andr (*p*< 0.05). Meanwhile, the FCR of 0.5 g/kg Andr group was 2.292 ± 0.064, which is significantly lower than the other group (*p*< 0.05). The SR of 0.5 g/kg and 1 g/kg Andr group were 85.000 ± 1.667%, 88.333 ± 0.076%, have no significantly difference with control group (*p*< 0.05). However, the SR of 2 g/kg Andr group was significantly lower than control group (**Table 1**, *p* > 0.05). The relationship between WG, SGR, FCR and Andr levels (**Figure 1**) was best expressed by the second-order polynomial regression equations as follows:

**Table 1:** Growth performance of *L. vannamei* fed diets supplemented with different levels of Andr for 4 weeks.

Diet	0 g/kg (Control)	0.5 g/kg	1 g/kg	2 g/kg
Initial weight	2.282 ± 0.082	2.198 ± 0.067	2.376 ± 0.151	2.578 ± 0.100
Final weight	4.354 ± 0.161	4.510 ± 0.140	4.414 ± 0.136	4.635 ± 0.318
WG (%)	81.592 ± 11.216	105.240 ± 6.245*	86.102 ± 7.620	79.949 ± 14.563
SGR (%)	2.204 ± 0.232	2.662 ± 0.113*	2.298 ± 0.149	2.168 ± 0.293
FCR	2.720 ± 0.495	2.292 ± 0.064*	2.528 ± 0.157	2.772 ± 0.282
SR (%)	83.333 ± 0.025	85.000 ± 1.667	88.333 ± 0.076	53.333 ± 0.104**

Andr, andrographolide; WG, weight gain rate; SGR, specific growth rate; FCR, Feed conversion ratio; SR, Survival rate. Values are means ± SD. * and ** means significantly difference compare with control group, * means p < 0.05, ** means p < 0.01.

$${\text{y}}^{\left(\text{WG}\right)}\;=\;-11.025{\text{x}}^{2}\;+\;47.738\text{x }+\;55.123$$

$${\text{y}}^{\left(\text{SGR}\right)}\;=\;-0.2144{\text{x}}^{2}\;+\;0.924\text{x }+\;1.6995$$

$${\text{y}}^{\text{(FCR)}}=\;0.2871{\text{x}}^{2}-1.2997\text{x}+3.6788$$

This figure showed that the optimum Andr level for *L. vannamei* is 0.5 g/kg diet.

### Effect of Andr on hemocytes phagocytic function of *L. vannamei*

**Figure 2:**
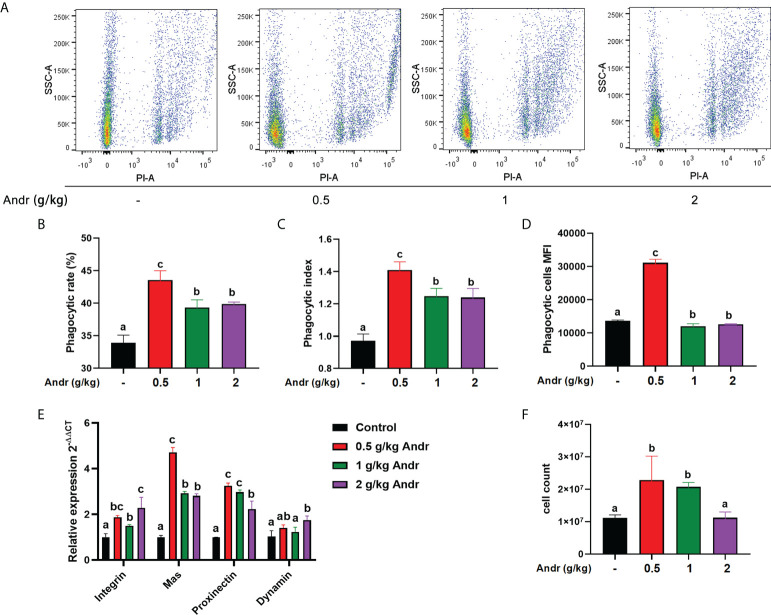
Effect of Andr on hemocytes phagocytic function of *L. vannamei*. **(A)** Scatter plot of phagocytic hemocytes ingested 0.5 µm microspheres. **(B–D)** The phagocytic rate, phagocytic index and phagocytic cells MFI in hemocytes. **(E)** The expression of phagocytic related genes in hemocytes detected by qPCR. **(F)** Total hemocytes count (THC) of *L. vannamei* fed a diet containing Andr for 28 days. Values are means ± SD. ^a,b,c^ Value bars not sharing the same superscript letter are significantly different as evaluated by Duncan ‘s test (*p*< 0.05).

The results of phagocytic function in hemocytes detected by flow cytometry were shown in **Figure 2A**. After fed shrimps with diet containing different concentration of Andr, the phagocytic rate and phagocytic index of hemocytes were significantly higher than the control group (**Figures 2B–D**, *p*< 0.05). The expression of phagocytic genes in hemocytes was consistent with flow cytometry (**Figure 2E**). Diet Andr significantly upregulated the expression of phagocytic associated gene Integrin, Mas, Proxinectin (*p*< 0.05). The gene expression of Integrin, Mas, Proxinectin were highest in the 0.5 g/kg Andr group, and then gradually decreased. In addition, the THC was significantly higher in shrimps fed 0.5 g/kg and 1 g/kg Andr compared with control group (**Figure 2F**, *p*< 0.05).

### Effect of Andr on immune-related gene of *L. vannamei*

**Figure 3:**
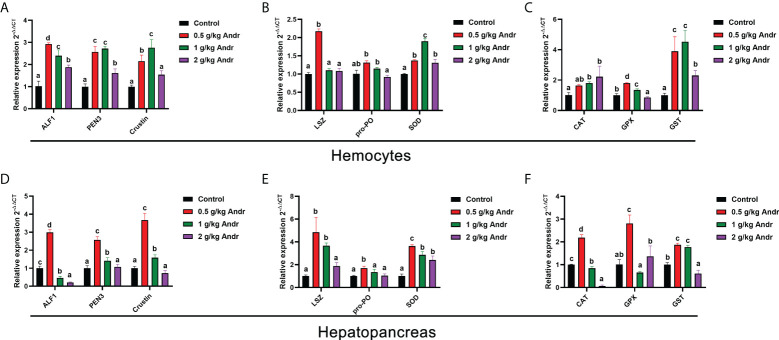
Effect of Andr on Immune-related gene in hemocyte and hepatopancreas of *L. vannamei.* **(A, D)** antimicrobial peptides; **(B, E)** immune functional proteins; **(C, F)** antioxidant protein. ALF1, Anti-lipopolysaccharide factor 1; PEN3, Penaeidin-3; LSZ, lysozyme; pro-PO, phenol oxidase; SOD, superoxide dismutase; CAT, catalase; GPX, glutathione peroxidase; GST, Glutathione S-transferase. Values are means ± SD. ^a,b,c,d^ Value bars not sharing the same superscript letter are significantly different as evaluated by Duncan ‘s test (*p*< 0.05).

To further explore the role of Andr on immunity of *L. vannamei*, expression of a series of immune related genes after fed the Andr for 4-week *in vivo* was investigated using RT-qPCR method, shown in **Figure 3**. Feeding diets supplemented with 0.5 g/kg Andr expression of almost all the examined immune effector genes involved in humoral immunity, including immune functional proteins (LSZ, PO and SOD), antimicrobial peptides (ALF1, PEN3 and crustin) and antioxidant protein (GST, GPX and CAT) significantly increased whether in hemocytes or hepatopancreas (**Figures 3A–F**, *p*< 0.05).

### Antioxidative parameters

**Figure 4:**
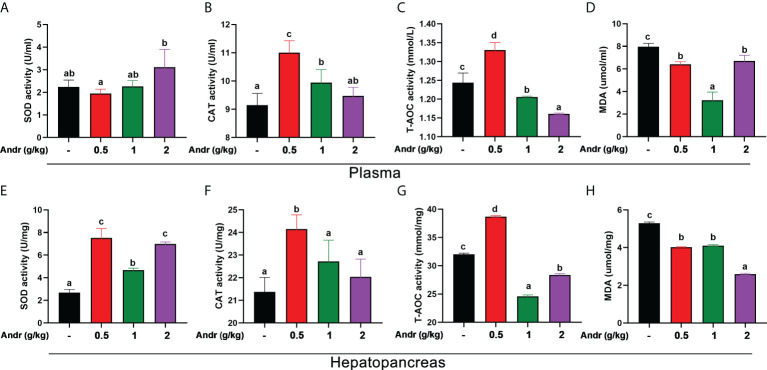
Effects of dietary Andr on antioxidative indices in plasma and hepatopancreas of *L.vannamei.* **(A, E)** SOD, total superoxide dismutase; **(B, F)** CAT, catalase; **(C, G)** T-AOC, total anti-oxidation capacity; **(D, H)** MDA, malondialdehyde. Values are means ± SD. ^a,b,c,d^ Value bars not sharing the same superscript letter are significantly different as evaluated by Duncan ‘s test (*p*< 0.05).

Dietary Andr significantly enhanced the SOD activity in plasma and hepatopancreas of *L. vannamei* (**Figures 4A, E**, *p*< 0.05). However, there was no significant difference in SOD activity between 0.5 g/kg and 1 g/kg Andr groups and the control group (*p* > 0.05). The capacity of T-AOC and the activity of CAT increased initially and decreased afterwards with increasing dietary Andr levels, which was significantly higher in shrimps fed 0.5 g/kg Andr group compared with control group (**Figures 4B, C, F, G**, *p*< 0.05). The MDA content in hepatopancreas was progressively decreased with the increase of dietary Andr, which had significantly lower values in 0.5 g/kg, 1 g/kg, and 2 g/kg Andr group compared with control group (**Figure 4D**, *p*< 0.05). The content of MDA in plasma of shrimps fed Andr group was significantly lower than that of control group (**Figure 4H**, *p*< 0.05), but the content of MDA was the lowest in 1 g/kg Andr group and slightly increased in 2 g/kg group.

### Andr alleviates oxidative damage and inflammatory response induced by *V. alginolyticus* infection on *L. vannamei*

**Figure 5:**
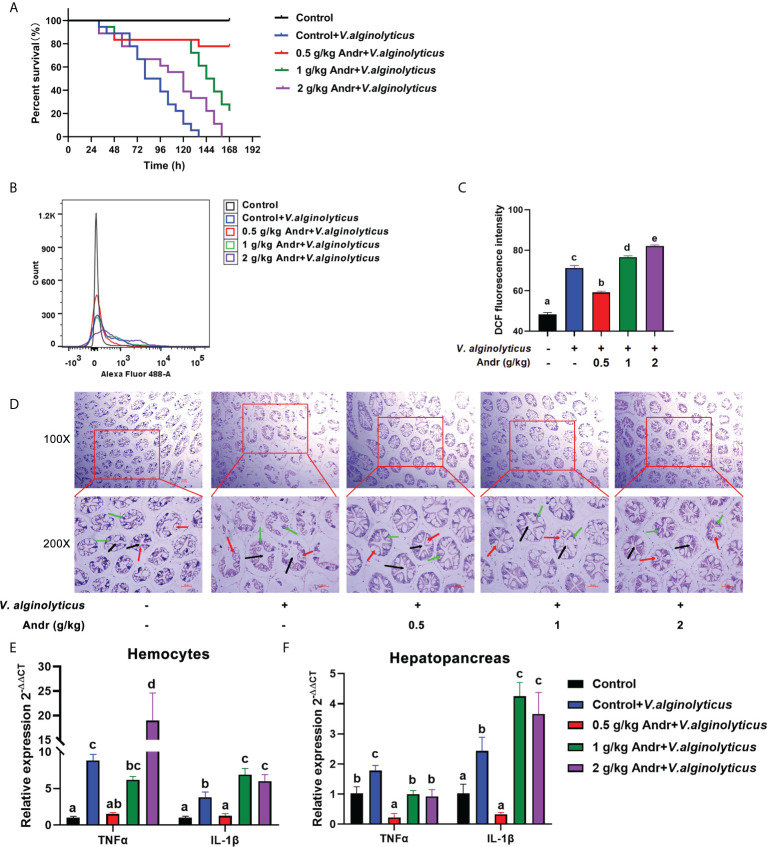
Andr alleviates oxidative damage and inflammatory response induced by *V. alginolyticus* infection on *L. vannamei*. **(A)** Percent survival of *L. vannamei* fed diet with Andr challenged with *V. alginolyticus*. **(B, C)** The ROS level in hemocytes was evaluated by analyzing DCF fluorescence intensity by flow cytometry. **(D)** Histopathological examination of the *L. vannamei* hepatopancreas under *V. alginolyticus* infection 6 h. Black arrows represent lumen of hepatic tubule of hepatopancreas, green arrows represent secretory cells of hepatopancreas, and red arrows represent absorptive cells of hepatopancreas. Scale bar, 50 mm. **(E, F)** The expression of TNFα and IL-1β detected by qPCR. Values are means ± SD. ^a,b,c,d,e^ Value bars not sharing the same superscript letter are significantly different as evaluated by Duncan ‘s test (*p*< 0.05).

In order to study the effect of Andr on *V. alginolyticus* infection of *L.vannamei*, *V. alginolyticus* challenge test was carried out on shrimps fed with Andr for 28 days (**Figure 5**). The results showed that the survival rate of shrimps fed contain Andr was significantly higher than that of control group after *V. alginolyticus* infection (*p*< 0.05). The survival rates of the control, 0.5 g/kg, 1 g/kg, 2g/kg Andr group were 0%, 77.7%, 22.2% and 0% at 7 days after *V. alginolyticus* infection, respectively (**Figure 5A**). The ROS levels of hemocytes in *L. vannamei* was detected by flow cytometry to investigate the effects of Andr on the oxidative damage of *L. vannamei* induced by *V. alginolyticus* infection. The result indicated that the generation of ROS in hemocytes of shrimps fed with Andr group were lower than that in control group (*p*< 0.05) (**Figures 5B, C**). Then, the damage of the hepatopancreas of shrimps was observed by histomorphology after *V. alginolyticus* infection. The results showed that the hepatopancreas of shrimps appeared fragmentation and hepatic tubular cavitation after *V. alginolyticus* infection. Feeding a diet containing Andr can alleviate hepatopancreas injury caused by *V. alginolyticus* infection (**Figure 5D**). Concurrently, the gene expression of TNFα and IL-1β in hemocytes and hepatopancreas were measured. The expression of inflammatory factors TNFα and IL-1β in hemocytes and hepatopancreas of shrimps were significantly up-regulated after *V. alginolyticus* infection. Feeding 0.5 g/kg Andr significantly inhibited the up-regulation of inflammatory factors induced by *V. alginolyticus* (**Figures 5E, F**, *p*< 0.05). However, the addition of 1 g/kg and 2 g/kg Andr showed no anti-inflammatory effect in hemocytes (*p*< 0.05).

### Andr inhibits cell apoptosis induced by *V. alginolyticus* infection on *L. vannamei*

**Figure 6:**
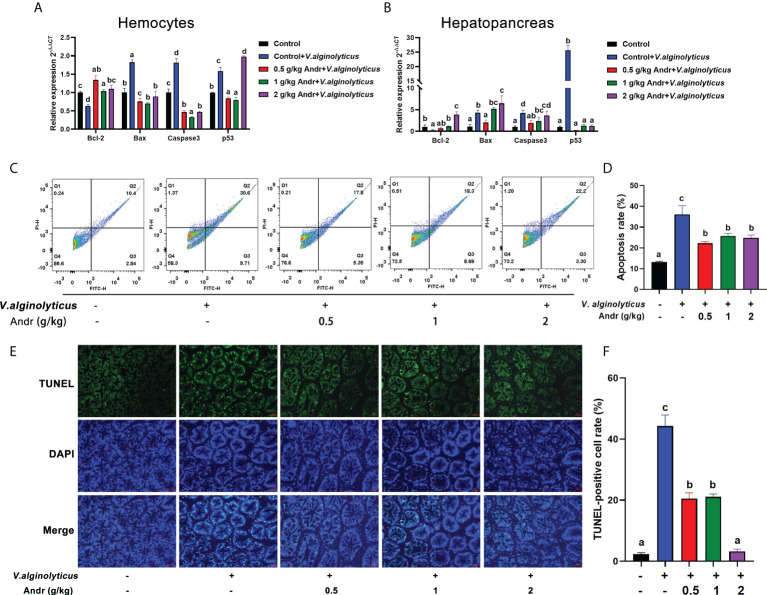
Andr inhibits cell apoptosis induced by *V. alginolyticus* infection on *L. vannamei*. **(A, B)** The expression of apoptosis-related gene in hemocytes and hepatopancreas of *L. vannamei* under *V. alginolyticus* infection 6 h. **(C, D)** Detection of apoptosis in hemocytes of *L. vannamei* by flow cytometry after *V. alginolyticus* infection 6 h. **(E, F)** Detection of apoptosis in hepatopancreas of *L. vannamei* by TUNEL stain after *V. alginolyticus* infection 6 h. Values are means ± SD. ^a,b,c,d^ Value bars not sharing the same superscript letter are significantly different as evaluated by Duncan ‘s test (*p*< 0.05).

The expression of apoptosis-related gene in hemocytes and hepatopancreas was detected by qRT-PCR (**Figures 6A, B**). The expression of pro-apoptotic genes (Bax, caspase3 and p53) was significantly up-regulated in hemocytes and hepatopancreas of shrimps infected with *V. alginolyticus* (*p*< 0.05). Compare with control group, the expression of pro-apoptotic genes (Bax, caspase3 and p53) in shrimps fed with 0.5 g/kg and 1 g/kg Andr was significantly decreased (*p*< 0.05). The anti-apoptotic gene Bcl-2 showed an opposite trend to the pro-apoptotic gene, and the difference was significant (*p*< 0.05). To further verify the effect of Andr on the apoptosis of shrimp induced by *V. alginolyticus*, flow cytometry was used to detect the apoptosis of hemocytes in shrimp (**Figure 6C**). After *V. alginolyticus* infection, the apoptosis rate of shrimps fed with Andr (0.5 g/kg, 1 g/kg and 2 g/kg) was significantly decreased than that in control group (**Figure 6D**, both *p*< 0.05). Similarly, TUNEL positive cell rate in shrimp hepatopancreas revealed that apoptotic cells increased after *V. alginolyticus* infection. Compare with control group, the rate of TUNEL-positive cells in hepatopancreas of shrimps fed with Andr was significantly reduced (**Figures 6E, F**, *p*< 0.05).

### Andr inhibited JNK activation induced by *V. alginolyticus* infection on *L. vannamei*

**Figure 7:**
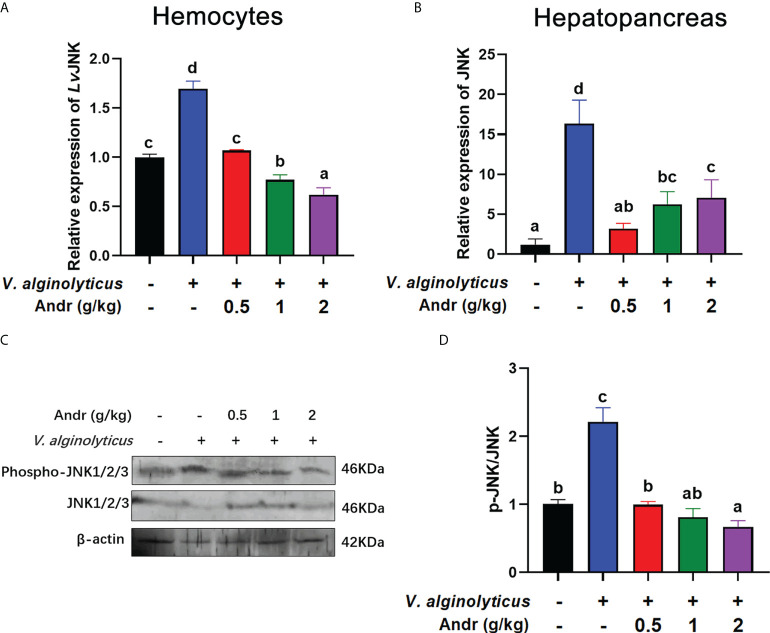
Andr inhibited JNK activation induced by *V. alginolyticus* infection on *L. vannamei*. **(A, B)** The gene expression of JNK in hemocytes and hepatopancreas detected by qPCR. **(C, D)** The expressions of JNK, p-JNK in hepatopancreas were analyzed by Western blot. Values are means ± SD. ^a,b,c,d^ Value bars not sharing the same superscript letter are significantly different as evaluated by Duncan ‘s test (*p*< 0.05).

Considering that JNK is important mediators of apoptosis, we examined the influence of Andr on JNK activity during *V. alginolyticus* infection. qRT-PCR analysis revealed an increase in the expressions of JNK whether in hemocytes or in hepatopancreas of shrimp after *V. alginolyticus* infection, indicating that JNK was activated by *V. alginolyticus* infection (**Figures 7A, B**, both *p*< 0.05). Fed shrimps with Andr mitigated this increase induced by *V. alginolyticus* (**Figures 7A, B**, both *p*< 0.05). Further study by western blot analysis found that *V. alginolyticus* infection could induce phosphorylation of JNK, while Andr could inhibit such phosphorylation (**Figures 7C, D**, both *p*< 0.05).

### Andr reduces *V. alginolyticus*-induced apoptosis by inhibiting ROS generation and JNK activation

**Figure 8:**
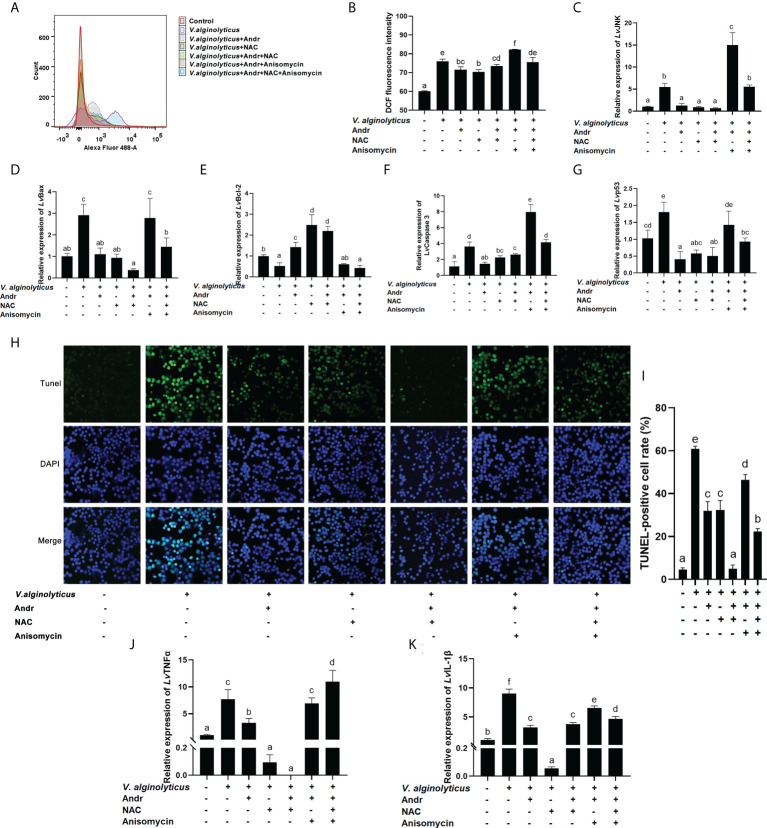
Andr reduces *V. alginolyticus*-induced apoptosis by inhibiting ROS generation and JNK activation. Uninfected or *V. alginolyticus*-infected cells were treated with Andr, NAC or anisomycin. At 24 h, the cells were detected for the related indices. **(A, B)** The ROS level were detected by flow cytometry. **(C-G)** The expression of JNK and apoptosis-related gene detected by qPCR. **(H, I)** The apoptosis detected by TUNEL stain. **(J, K)** The expression of TNFα and IL-1β detected by qPCR. Values are means ± SD. ^a,b,c,d,e,f^ Value bars not sharing the same superscript letter are significantly different as evaluated by Duncan ‘s test (*p*< 0.05).

To further clarify the mechanism of Andr alleviating *V. alginolyticus*-induced apoptosis from the perspective of JNK and ROS, we treated hemocytes *in vitro* with the JNK specific inhibitor anisomycin and the ROS scavenger NAC, respectively. Then, the generation of ROS, the rate of apoptosis, the expressions of apoptosis-related genes and inflammatory factor was analyzed. The generation of ROS induced by *V. alginolyticus* was detected by DCFH-DA staining and flow cytometry analysis. Compared with the control group, DCFH-DA fluorescence intensity *in vitro* was dramatically stimulated by *V. alginolyticus*. The Andr obviously weakened the production of ROS in hemocytes treated with *V. alginolyticus*, with the same effect observed in the group treated with NAC (**Figures 8A, B**, *p*< 0.05). Treat with anisomycin, the attenuating effect of Andr and NAC on the ROS production of hemocytes under *V. alginolyticus* infection disappeared (*p*< 0.05). Meanwhile, the gene expression of JNK was significantly suppressed by Andr and NAC, and activated by anisomycin (**Figure 8C**, *p*< 0.05). Furthermore, changes in cell apoptosis were detected by TUNEL-stain assay to investigate the mechanism of Andr on the apoptosis induced by *V. alginolyticus*. The TUNEL-positive rate was significantly elevated in response to *V. alginolyticus* treatment, which could be reversed by treatment with NAC. The addition of Andr also substantially decreased the TUNEL-positive rate, similar to NAC treatment (**Figures 8H, I**, *p*< 0.05). Consistent with the result of TUNEL stain, the up-regulation of pro-apoptosis genes (Bax, caspase 3 and p53) and the down-regulation of anti-apoptosis genes (Bcl-2) induced by *V. alginolyticus* was suppressed by Andr and NAC (**Figures 8C–G**, *p*< 0.05). Moreover, we further verified whether Andr could inhibit the *V. alginolyticus* -induced activation of inflammation through ROS. As illustrated in **Figures 8J, K**, Andr and NAC both clearly reversed the over expression of TNFα and IL-1β induced by *V. alginolyticus* stimulation (*p*< 0.05). These results indicated that *V. alginolyticus* could activate JNK and the apoptosis signaling pathways by inducing ROS overproduction. Meanwhile, Andr could inhibit the above inflammatory response through ROS-JNK signaling.

## Discussion

Bacterial infection is an important biological factor restricting the development of shrimp culture. *V. alginolyticus* is one of the major pathogens causing mass mortality of shrimps worldwide, affecting energy metabolism, immune response and development of shrimps (52–54). Our previous studies have shown that the infection of *V. alginolyticus* can affect the survival, growth and metabolism of shrimp, induce the production of ROS, apoptosis and autophagy, reduce immune function, and cause DNA damage (22, 55–57). Therefore, it is necessary to find a drug that can protect shrimp from *V. alginolyticus*. Andr has been reported to be a promising therapeutic for treatment of multiple types of infectious diseases, with anti-inflammatory, antioxidant, and immunomodulatory functions (58). However, little is known about the effects and biochemical mechanisms of action of Andr in the infection of *V. alginolyticus* on *L. vannamei*. In the present study, the results demonstrated that Andr promote the growth and immunity of *L. vannamei*, and protects shrimp against *V. alginolyticus* by regulating inflammation and apoptosis *via* a ROS-JNK dependent pathway.

Andr being used as a dietary supplement to improve immunity in patients has a long history (48, 59, 60). In recent years, Andr has also been used as feed additive in animal husbandry (61) and aquaculture (49, 50). In the present study, *L. vannamei* fed diets with 0.5 g/kg Andr showed significantly higher WG and SGR than shrimps fed the diet with 0 g/kg, 1 g/kg and 2 g/kg Andr. Consistent with many plants extract additive studies, the weight gain of shrimps first increased and then decreased with increasing amounts of additive (25). In addition, the addition amount of Andr varies greatly among different species. In *L. rohita*, a higher weight gain (BWG) and protein efficiency ratio (PER) was found when fed a diet supplemented with Andr (1 -16 g/kg feed) (49). In *M.albus*, the optimal supplemental level of Andr was 75-150 mg/kg (50), which was far lower than that of *L. rohita* and shrimp in the present study. Several studies showed strong effects of plants in boosting the shrimp immune system against pathogens in aquaculture (25, 62). In Crustaceans, THC, phenoloxidase activity, antioxidant enzyme activity, antimicrobial peptide and phagocytic activity are commonly considered as useful indicators of immunosurveillance status in animals (24). It has been shown that the addition of Andr to fish diets can increase phagocytic activity of hemocyte (49). In line with the previous findings, dietary Andr enhanced the THC and phagocytosis of shrimps hemocytes. Like hemocytes, hepatopancreas are also important immune organs in crustaceans, which play a very important role in the immune response against pathogen invasion (22). Results obtained in the present study showed that dietary Andr significantly upregulated the expression of immune-related genes, antimicrobial peptide genes, antioxidant genes and enzyme activity in both hemocytes and hepatopancreas. A similar result was found that Andr increased the activities of SOD, CAT, GPx, GST, GSH and GR in the liver (50). Considering these indicators together, we concluded that Andr promoted the immunity of shrimp organism. In this study, dietary Andr also significantly enhanced the resistance of shrimp to *V. alginolyticus*, which directly reflected the improvement of immune function by Andr.

ROS can function as key indicators that serve a prominent role in the mediation of both cell survival and death following exposure to various stimuli (63). Increased ROS can induce oxidative stress when the balance between oxidation and reduction-regulated cellular processes is disrupted and cells are unable to repair the resulting oxidative damage (64). Our previous studies demonstrated that the increased ROS production and DNA damage coexisted in hemocytes at 6 h after *V. alginolyticus* infection (55, 65). Thus, controlling the cellular ROS levels is conducive to the survival of cells in adverse environment (66, 67). Andr has been reported to ameliorate damage caused by disease by reducing oxidative stress and inflammation (35, 68). Herein, Andr treatment significantly reduce the increase of ROS production and inflammation in shrimp hemocytes induced by *V. alginolyticus* infection, indicating that Andr ameliorates oxidative stress and inflammation in shrimp hemocytes induced by *V. alginolyticus* infection. Excessive amounts of ROS can affect multiple signaling pathways, such as MAPKs, JNK, NF-KB, result in oxidative stress, excessive inflammatory responses, tissue damage and apoptosis (20, 69). For extracellular bacteria such as *V, alginolyticus*, apoptosis induction in host cells is not necessarily associated with direct bacterial contact. Rather, various cytokines produced by the immune system plays major roles in apoptosis-induction and sensitivity (70). *V. alginolyticus* regulates proinflammatory cytokines IL-1β, IL-6, IL-12 and TNFα production in macrophages (5) accompanied by the activation of JNK pathways (71). Both endogenous and endogenous ROS induce JNK activation (72), which plays a key role in inducing apoptosis (73). The JNK pathway represents one sub-group of MAP kinases that activated by a series of phosphorylation in response to various stress stimuli like environmental stresses and inflammatory cytokines (74–76). The inhibitors of JNK (SP600125) significantly reduced the production of IL-1β, IL-6, IL-12 and TNFα induced by *V. alginolyticus* (71). Therefore, inhibition of JNK signaling may be a perfect adjuvant therapy for *V. alginolyticus* infections. Several studies have demonstrated that the phosphorylation of MAPKs is mediated by Andr (77–79). Treatment with Andr significantly increased the expression levels of phosphorylated JNK in osteosarcoma cells (80). In this study, we also observed that *V. alginolyticus* infection could lead to increased JNK activation and apoptosis in hepatopancreas of *L. vannamei*, while Andr could inhibit this increase.

Considering the role of ROS production and JNK activation in triggering apoptosis and inflammation, we hypothesized that Andr inhibits the activation of JNK pathway by excessive ROS, which may be one of the reasons that Andr protects *L. vannamei* from *V. alginolyticus* infection. Thereafter, we investigated the mechanism of Andr alleviates *V. alginolyticus*-induced inflammation and apoptosis from the perspective of ROS and JNK activation. The ROS scavenger NAC was used to inhibit ROS production *in vitro*. The results showed that the ROS scavenger NAC decreased the expression of apoptotic and inflammation genes (Bax, caspase3, p53 and IL-1β, TNFα) and the TUNEL-positive cell rate induced by *V. alginolyticus* infection, ascertaining that ROS acts as an upstream signal in *V. alginolyticus* induced apoptosis. Andr can mitigate ROS generation and thus reduce the inflammation and apoptosis caused by *V. alginolyticus*. Intriguingly, the inhibitory effect of Andr on ROS generation was similar to that of NAC. Furthermore, we activated the JNK signaling pathway through anisomycin on the basis of Andr or NAC treatment of cells. Anisomycin counteracts the inhibitory effect of Andr or NAC on *V. alginolyticus* induced inflammation and apoptosis, implicating that Andr inhibits *V. alginolyticus*-induced inflammation and apoptosis by inhibiting the activation of JNK pathway.

**Figure 9:**
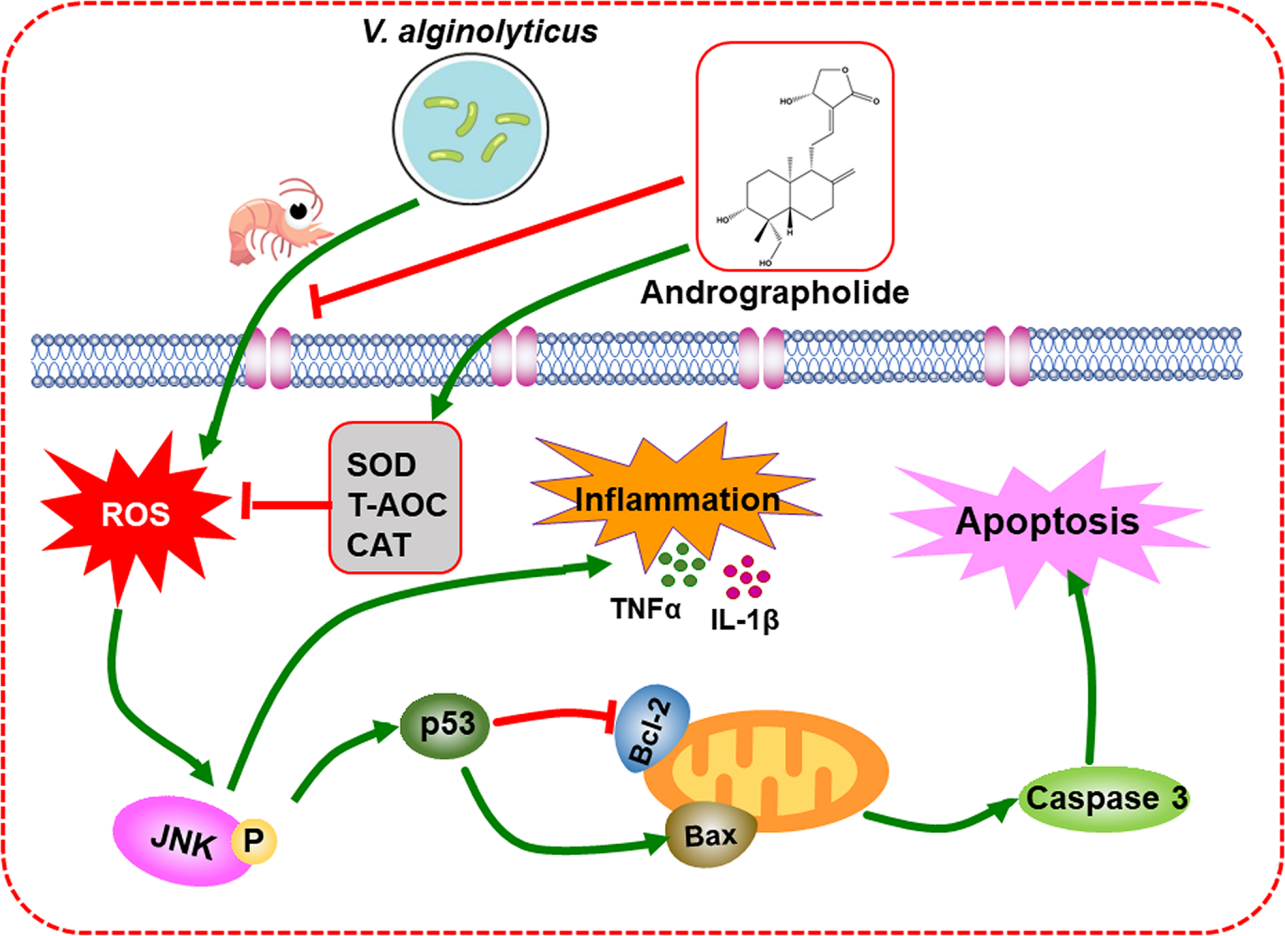
Schematic representation of the inhibitory mechanism of Andr on *V. alginolyticus*-induced inflammation and apoptosis.

In conclusion, the present study showed that the supplementation of Andr in diets significantly enhanced the growth and promoted the non-specific immunity and the resistance to *V. alginolyticus* in *L. vannamei*. Furthermore, a mechanism of Andr protects *L. vannamei* against *V. alginolyticus* was certified in the present study. Andr inhibited the inflammation and apoptosis induced by *V. alginolyticus via* a ROS-JNK dependent pathway (**Figure 9**). These results improve the understanding of the pathogenesis of *V. alginolyticus* infection and provide clues to the development of effective drugs against *V. alginolyticus*.

## Data availability statement

The original contributions presented in the study are included in the article/**Supplementary Material**. Further inquiries can be directed to the corresponding author.

## Ethics statement

The animal study was reviewed and approved by South China Normal University.

## Author contributions

XY and WW: Conceptualization, Methodology, Software Data curation, Writing- Original draft preparation. XZ and WL: Visualization, Investigation, Software, Validation. ML and LH: Visualization, Investigation. QC, JH, CY and ZJ: Shrimp culture, Methodology, Supervision. YL and WW: Writing- Reviewing and Editing. All authors contributed to the article and approved the submitted version.

## Funding

This work was supported by the National Nature Science Foundation of China (Grant No.31971417) and Guangdong Province Natural Science Foundation of China (Grant No. 2019A1515011442).

## Conflict of interest

The authors declare that the research was conducted in the absence of any commercial or financial relationships that could be construed as a potential conflict of interest.

## Publisher’s note

All claims expressed in this article are solely those of the authors and do not necessarily represent those of their affiliated organizations, or those of the publisher, the editors and the reviewers. Any product that may be evaluated in this article, or claim that may be made by its manufacturer, is not guaranteed or endorsed by the publisher.

## Supplementary material

Supplementary Figure 1 CCK8 assay was used to determine the concentrations of N-acetyl-l-cysteine (NAC) and anisomycin.

## References

[1] Wang Z Wang B Chen G Jian J Lu Y Xu Y. Transcriptome analysis of the pearl oyster (*Pinctada fucata*) hemocytes in response to *Vibrio alginolyticus* infection. Gene (2016) 2(575):421–8. doi: 10.1016/j.gene.2015.09.014 26363408

[2] Mohamad N Mohd Roseli FA Azmai MNA Saad MZ Md Yasin IS Zulkiply NA. Natural concurrent infection of *Vibrio harveyi* and *V. alginolyticus* in cultured hybrid groupers in Malaysia. J Aquat Anim Health (2019) 31(1):88–96. doi: 10.1002/aah.10055 30536485

[3] Liao G Wu Q Mo B Zhou J Li J Zou J. Intestinal morphology and microflora to *Vibrio alginolyticus* in pacific white shrimp (Litopenaeus vannamei). Fish Shellfish Immunol (2022) 121:437–45. doi: 10.1016/j.fsi.2022.01.026 35065276

[4] Jacobs Slifka KM Newton AE Mahon BE. *vibrio alginolyticus* infections in the USA, 1988-2012. Epidemiol Infect (2017) 145(7):1491–9. doi: 10.1017/s0950268817000140 PMC9203320 28202099

[5] Wang J Ding Q Yang Q Fan H Yu G Liu F. *Vibrio alginolyticus* triggers inflammatory response in mouse peritoneal macrophages *Via* activation of Nlrp3 inflammasome. Front Cell Infect Microbiol (2021) 11:769777. doi: 10.3389/fcimb.2021.769777 34869071 PMC8634873

[6] Osunla CA Okoh AI. Vibrio pathogens: A public health concern in rural water resources in Sub-Saharan Africa. Int J Environ Res Public Health (2017) 14(10):1188. doi: 10.3390/ijerph14101188 PMC5664689 28991153

[7] Oberbeckmann S Wichels A Wiltshire KH Gerdts G. Occurrence of vibrio parahaemolyticus and *Vibrio alginolyticus* in the German bight over a seasonal cycle. Antonie Van Leeuwenhoek (2011) 100(2):291–307. doi: 10.1007/s10482-011-9586-x 21598011

[8] Nguyen HT Van TN Ngoc TT Boonyawiwat V Rukkwamsuk T Yawongsa A. Risk factors associated with acute hepatopancreatic necrosis disease at shrimp farm level in bac lieu province, Vietnam. Veterinary World (2021) 14(4):1050–8. doi: 10.14202/vetworld.2021.1050-1058 PMC8167526 34083959

[9] Nunan L Lightner D Pantoja C Gomez-Jimenez S. Detection of acute hepatopancreatic necrosis disease (Ahpnd) in Mexico. Dis Aquat Organisms (2014) 111(1):81–6. doi: 10.3354/dao02776 25144120

[10] Chen MX Li HY Li G Zheng TL. Distribution of *Vibrio alginolyticus*-like species in shenzhen coastal waters, China. Braz J Microbiol [publication Braz Soc Microbiol] (2011) 42(3):884–96. doi: 10.1590/s1517-83822011000300007 PMC3768764 24031704

[11] Zanetti S Deriu A Volterra L Falchi MP Molicotti P Fadda G. Virulence factors in *Vibrio alginolyticus* strains isolated from aquatic environments. Annali Di Igiene Med Preventiva Di Comunita (2000) 12(6):487–91. 11235505

[12] Wang Q Liu Q Cao X Yang M Zhang Y. Characterization of two tonb systems in marine fish pathogen *Vibrio alginolyticus*: Their roles in iron utilization and virulence. Arch Microbiol (2008) 190(5):595–603. doi: 10.1007/s00203-008-0407-1 18629473

[13] Zhao Z Chen C Hu CQ Ren CH Zhao JJ Zhang LP. The type iii secretion system of *Vibrio alginolyticus* induces rapid apoptosis, cell rounding and osmotic lysis of fish cells. Microbiol (Reading England) (2010) 156(Pt 9):2864–72. doi: 10.1099/mic.0.040626-0 20576689

[14] Zhao Z Liu J Deng Y Huang W Ren C Call DR. The *Vibrio alginolyticus* T3ss effectors, Val1686 and Val1680, induce cell rounding, apoptosis and lysis of fish epithelial cells. Virulence (2018) 9(1):318–30. doi: 10.1080/21505594.2017.1414134 PMC5955196 29252102

[15] Hernández-Robles MF Álvarez-Contreras AK Juárez-García P Natividad-Bonifacio I Curiel-Quesada E Vázquez-Salinas C. Virulence factors and antimicrobial resistance in environmental strains of *Vibrio alginolyticus* . Int Microbiol Off J Spanish Soc Microbiol (2016) 19(4):191–8. doi: 10.2436/20.1501.01.277 28504816

[16] Gu D Zhang J Hao Y Xu R Zhang Y Ma Y. Alternative sigma factor rpox is a part of the rpoe regulon and plays distinct roles in stress responses, motility, biofilm formation, and hemolytic activities in the marine pathogen *Vibrio alginolyticus* . Appl Environ Microbiol (2019) 85(14):e00234-19. doi: 10.1128/aem.00234-19 31053580 PMC6606886

[17] Bunpa S Chaichana N Teng JLL Lee HH Woo PCY Sermwittayawong D. Outer membrane protein a (Ompa) is a potential virulence factor of *Vibrio alginolyticus* strains isolated from diseased fish. J Fish Dis (2020) 43(2):275–84. doi: 10.1111/jfd.13120 31779054

[18] Chen YH He JG. Effects of environmental stress on shrimp innate immunity and white spot syndrome virus infection. Fish Shellfish Immunol (2019) 84:744–55. doi: 10.1016/j.fsi.2018.10.069 30393174

[19] Kaiser P Hardt WD. Salmonella typhimurium diarrhea: Switching the mucosal epithelium from homeostasis to defense. Curr Opin Immunol (2011) 23(4):456–63. doi: 10.1016/j.coi.2011.06.004 21726991

[20] Dixon SJ Stockwell BR. The role of iron and reactive oxygen species in cell death. Nat Chem Biol (2014) 10(1):9–17. doi: 10.1038/nchembio.1416 24346035

[21] Zhang B Liu SQ Li C Lykken E Jiang S Wong E. Microrna-23a curbs necrosis during early T cell activation by enforcing intracellular reactive oxygen species equilibrium. Immunity (2016) 44(3):568–81. doi: 10.1016/j.immuni.2016.01.007 PMC4794397 26921109

[22] Kong JR Wei W Liang QJ Qiao XL Kang H Liu Y. Identifying the function of Lvpi3k during the pathogenic infection of litopenaeus vannamei by *vibrio alginolyticus* . Fish Shellfish Immunol (2018) 76:355–67. doi: 10.1016/j.fsi.2018.03.016 29544772

[23] Mao F Liu K Wong NK Zhang X Yi W Xiang Z. Virulence of *vibrio alginolyticus* accentuates apoptosis and immune rigor in the oyster crassostrea hongkongensis. Front Immunol (2021) 12:746017. doi: 10.3389/fimmu.2021.746017 34621277 PMC8490866

[24] Matozzo V Marin MG. The role of haemocytes from the crab carcinus aestuarii (Crustacea, decapoda) in immune responses: A first survey. Fish Shellfish Immunol (2010) 28(4):534–41. doi: 10.1016/j.fsi.2009.12.003 20036746

[25] Dewi NR Huang HT Wu YS Liao ZH Lin YJ Lee PT. Guava (Psidium guajava) leaf extract enhances immunity, growth, and resistance against vibrio parahaemolyticus in white shrimp penaeus vannamei. Fish Shellfish Immunol (2021) 118:1–10. doi: 10.1016/j.fsi.2021.08.017 34418559

[26] Xie JJ Chen X Guo TY Xie SW Fang HH Liu ZL. Dietary values of forsythia suspensa extract in penaeus monodon under normal rearing and vibrio parahaemolyticus 3hp (Vp(3hp)) challenge conditions: Effect on growth, intestinal barrier function, immune response and immune related gene expression. Fish Shellfish Immunol (2018) 75:316–26. doi: 10.1016/j.fsi.2018.02.030 29454898

[27] Sun Z Tan X Wei Z Liu Q Mai H Liu Y. Effects of dietary dandelion extract on the growth performance, serum biochemical parameters, liver histology, and immune and apoptosis-related genes expression of hybrid grouper (Epinephelus lanceolatus♂ × epinephelus fuscoguttatus♀) at different feeding period. Fish Shellfish Immunol (2022) 120:280–6. doi: 10.1016/j.fsi.2021.11.034 34838703

[28] Cirmi S Randazzo B Russo C Musumeci L Maugeri A Montalbano G. Anti-inflammatory effect of a flavonoid-rich extract of orange juice in adult zebrafish subjected to vibrio anguillarum-induced enteritis. Nat Prod Res (2021) 35(23):5350–3. doi: 10.1080/14786419.2020.1758096 32338069

[29] Chakravarti RN Chakravarti D. Andrographolide, the active constituent of andrographis paniculata nees; a preliminary communication. Indian Med Gazette (1951) 86(3):96–7. PMC5191793 14860885

[30] Choudhury BR Poddar MK. Andrographolide and kalmegh (Andrographis paniculata) extract: *In vivo* and *in vitro* effect on hepatic lipid peroxidation. Methods Findings Exp Clin Pharmacol (1984) 6(9):481–5. 6513681

[31] Wintachai P Kaur P Lee RC Ramphan S Kuadkitkan A Wikan N. Activity of andrographolide against chikungunya virus infection. Sci Rep (2015) 5:14179. doi: 10.1038/srep14179 26384169 PMC4585663

[32] Guo X Zhang LY Wu SC Xia F Fu YX Wu YL. Andrographolide interferes quorum sensing to reduce cell damage caused by avian pathogenic escherichia coli. Veterinary Microbiol (2014) 174(3-4):496–503. doi: 10.1016/j.vetmic.2014.09.021 25448450

[33] Chua LS. Review on liver inflammation and antiinflammatory activity of andrographis paniculata for hepatoprotection. Phytother Res PTR (2014) 28(11):1589–98. doi: 10.1002/ptr.5193 25043965

[34] Zhang C Qiu X. Andrographolide radiosensitizes human ovarian cancer Skov3 xenografts due to an enhanced apoptosis and autophagy. Tumour Biol J Int Soc Oncodevelopmental Biol Med (2015) 36(11):8359–65. doi: 10.1007/s13277-015-3578-9 26014516

[35] Peng S Gao J Liu W Jiang C Yang X Sun Y. Andrographolide ameliorates ova-induced lung injury in mice by suppressing ros-mediated nf-κb signaling and Nlrp3 inflammasome activation. Oncotarget (2016) 7(49):80262–74. doi: 10.18632/oncotarget.12918 PMC5348318 27793052

[36] Lim JC Chan TK Ng DS Sagineedu SR Stanslas J Wong WS. Andrographolide and its analogues: Versatile bioactive molecules for combating inflammation and cancer. Clin Exp Pharmacol Physiol (2012) 39(3):300–10. doi: 10.1111/j.1440-1681.2011.05633.x 22017767

[37] Dai Y Chen S-R Chai L Zhao J Wang Y Wang Y. Overview of pharmacological activities of andrographis paniculata and its major compound andrographolide. Crit Rev Food Sci Nutr (2019) 59(sup1):S17–29. doi: 10.1080/10408398.2018.1501657 30040451

[38] Tan WSD Liao W Zhou S Wong WSF. Is there a future for andrographolide to be an anti-inflammatory drug? deciphering its major mechanisms of action. Biochem Pharmacol (2017) 139:71–81. doi: 10.1016/j.bcp.2017.03.024 28377280

[39] Gupta S Mishra KP Singh SB Ganju L. Inhibitory effects of andrographolide on activated macrophages and adjuvant-induced arthritis. Inflammopharmacology (2018) 26(2):447–56. doi: 10.1007/s10787-017-0375-7 28735448

[40] Chiou WF Lin JJ Chen CF. Andrographolide suppresses the expression of inducible nitric oxide synthase in macrophage and restores the vasoconstriction in rat aorta treated with lipopolysaccharide. Br J Pharmacol (1998) 125(2):327–34. doi: 10.1038/sj.bjp.0702073 PMC1565624 9786505

[41] Zhu T Wang DX Zhang W Liao XQ Guan X Bo H. Andrographolide protects against lps-induced acute lung injury by inactivation of nf-κb. PloS One (2013) 8(2):e56407. doi: 10.1371/journal.pone.0056407 23437127 PMC3578846

[42] Lu CY Yang YC Li CC Liu KL Lii CK Chen HW. Andrographolide inhibits tnfα-induced icam-1 expression *Via* suppression of nadph oxidase activation and induction of ho-1 and gclm expression through the Pi3k/Akt/Nrf2 and Pi3k/Akt/Ap-1 pathways in human endothelial cells. Biochem Pharmacol (2014) 91(1):40–50. doi: 10.1016/j.bcp.2014.06.024 24998495

[43] Zhang Q Liu J Duan H Li R Peng W Wu C. Activation of Nrf2/Ho-1 signaling: An important molecular mechanism of herbal medicine in the treatment of atherosclerosis *Via* the protection of vascular endothelial cells from oxidative stress. J Adv Res (2021) 34:43–63. doi: 10.1016/j.jare.2021.06.023 35024180 PMC8655139

[44] Lin HC Su SL Lu CY Lin AH Lin WC Liu CS. Andrographolide inhibits hypoxia-induced hif-1α-Driven endothelin 1 secretion by activating Nrf2/Ho-1 and promoting the expression of prolyl hydroxylases 2/3 in human endothelial cells. Environ Toxicol (2017) 32(3):918–30. doi: 10.1002/tox.22293 27297870

[45] Burgos RA Alarcón P Quiroga J Manosalva C Hancke J. Andrographolide, an anti-inflammatory multitarget drug: All roads lead to cellular metabolism. Mol (Basel Switzerland) (2020) 26(1):5. doi: 10.3390/molecules26010005 PMC7792620 33374961

[46] Geng J Liu W Gao J Jiang C Fan T Sun Y. Andrographolide alleviates parkinsonism in mptp-pd mice *Via* targeting mitochondrial fission mediated by dynamin-related protein 1. Br J Pharmacol (2019) 176(23):4574–91. doi: 10.1111/bph.14823 PMC6932945 31389613

[47] Malik Z Parveen R Parveen B Zahiruddin S Aasif Khan M Khan A. Anticancer potential of andrographolide from andrographis paniculata (Burm.F.) nees and its mechanisms of action. J Ethnopharmacol (2021) 272:113936. doi: 10.1016/j.jep.2021.113936 33610710

[48] Puri A Saxena R Saxena RP Saxena KC Srivastava V Tandon JS. Immunostimulant agents from andrographis paniculata. J Nat Prod (1993) 56(7):995–9. doi: 10.1021/np50097a002 8377022

[49] Basha KA Raman RP Prasad KP Kumar K Nilavan E Kumar S. Effect of dietary supplemented andrographolide on growth, non-specific immune parameters and resistance against aeromonas hydrophila in labeo rohita (Hamilton). Fish Shellfish Immunol (2013) 35(5):1433–41. doi: 10.1016/j.fsi.2013.08.005 23973382

[50] Shi Y Zhong L Liu Y Zhang J Lv Z Li Y. Effects of dietary andrographolide levels on growth performance, antioxidant capacity, intestinal immune function and microbioma of rice field eel (Monopterus albus). Anim Open Access J MDPI (2020) 10(10):1744. doi: 10.3390/ani10101744 PMC7599621 32992929

[51] Cárdenas JV Gálvez AO Brito LO Galarza EV Pitta DC Rubin VV. Assessment of different levels of green and brown seaweed meal in experimental diets for whiteleg shrimp (Litopenaeus vannamei, Boone) in recirculating aquaculture system. Aquacult Int (2015) 23(6):1491–504. doi: 10.1007/s10499-015-9899-2

[52] Chen Y Cai S Jian J. Protection against *vibrio alginolyticus* in pearl gentian grouper (♀Epinephelus fuscoguttatus × ♂Epinephelus lanceolatu) immunized with an acfa-deletion live attenuated vaccine. Fish Shellfish Immunol (2019) 86:875–81. doi: 10.1016/j.fsi.2018.12.030 30572128

[53] Kang CH Shin Y Jang S Jung Y So JS. Antimicrobial susceptibility of *Vibrio alginolyticus* isolated from oyster in Korea. Environ Sci pollut Res Int (2016) 23(20):21106–12. doi: 10.1007/s11356-016-7426-2 27543129

[54] Zhu F Qian X Ma X. Comparative transcriptomic analysis of crab hemocytes in response to white spot syndrome virus or *Vibrio alginolyticus* infection. Fish Shellfish Immunol (2018) 80:165–79. doi: 10.1016/j.fsi.2018.06.003 29870828

[55] Gu MM Kong JR Di H Peng T Xie CY Yang KY. Molecular characterization and function of the Prohibitin2 gene in litopenaeus vannamei responses to *Vibrio alginolyticus* . Dev Comp Immunol (2017) 67:177–88. doi: 10.1016/j.dci.2016.10.004 27756688

[56] Xie CY Kong JR Zhao CS Xiao YC Peng T Liu Y. Molecular characterization and function of a pten gene from litopenaeus vannamei after *Vibrio alginolyticus* challenge. Dev Comp Immunol (2016) 59:77–88. doi: 10.1016/j.dci.2016.01.004 26801100

[57] Wang F Huang L Liao M Dong W Liu C Liu Y. Integrative analysis of the mirna-mrna regulation network in hemocytes of penaeus vannamei following *vibrio alginolyticus* infection. Dev Comp Immunol (2022) 131:104390. doi: 10.1016/j.dci.2022.104390 35276318

[58] Vetvicka V Vannucci L. Biological properties of andrographolide, an active ingredient of andrographis paniculata: A narrative review. Ann Trans Med (2021) 9(14):1186. doi: 10.21037/atm-20-7830 PMC8350652 34430627

[59] Mishra A Shaik HA Sinha RK Shah BR. Andrographolide: A herbal-chemosynthetic approach for enhancing immunity, combating viral infections, and its implication on human health. Mol (Basel Switzerland) (2021) 26(22):7036. doi: 10.3390/molecules26227036 PMC8622020 34834128

[60] Naik SR Hule A. Evaluation of immunomodulatory activity of an extract of andrographolides from andographis paniculata. Planta Med (2009) 75(8):785–91. doi: 10.1055/s-0029-1185398 19263340

[61] Yusuf AL Adeyemi KD Roselina K Alimon AR Goh YM Samsudin AA. Dietary supplementation of different parts of andrographis paniculata affects the fatty acids, lipid oxidation, microbiota, and quality attributes of longissimus muscle in goats. Food Res Int (Ottawa Ont) (2018) 111:699–707. doi: 10.1016/j.foodres.2018.06.015 30007735

[62] Bussabong P Rairat T Chuchird N Keetanon A Phansawat P Cherdkeattipol K. Effects of isoquinoline alkaloids from macleaya cordata on growth performance, survival, immune response, and resistance to vibrio parahaemolyticus infection of pacific white shrimp (Litopenaeus vannamei). PloS One (2021) 16(5):e0251343. doi: 10.1371/journal.pone.0251343 33956913 PMC8101937

[63] Dickson KB Zhou J. Role of reactive oxygen species and iron in host defense against infection. Front Biosci (Landmark Ed) (2020) 25(8):1600–16. doi: 10.2741/4869 32114446

[64] Luderer U. Ovarian toxicity from reactive oxygen species. Vitamins Hormones (2014) 94:99–127. doi: 10.1016/b978-0-12-800095-3.00004-3 24388188

[65] Huang D Qiao X-L Q-j L Wei W Kong J-R Huan Kang C-s. Molecular characterization and function analysis of a nucleotide excision repair gene Rad23 from litopenaeus vannamei after *vibrio alginolyticus* challenge. Fish Shellfish Immunol (2018) 83:190–204. doi: 10.1016/j.fsi.2018.09.021 30195911

[66] Filomeni G De Zio D Cecconi F. Oxidative stress and autophagy: The clash between damage and metabolic needs. Cell Death Differentiation (2015) 22(3):377–88. doi: 10.1038/cdd.2014.150 PMC4326572 25257172

[67] Van Erp AC Hoeksma D Rebolledo RA Ottens PJ Jochmans I Monbaliu D. The crosstalk between ros and autophagy in the field of transplantation medicine. Oxid Med Cell Longevity (2017) 2017:7120962. doi: 10.1155/2017/7120962 PMC5749284 29410735

[68] Ji X Li C Ou Y Li N Yuan K Yang G. Andrographolide ameliorates diabetic nephropathy by attenuating hyperglycemia-mediated renal oxidative stress and inflammation *Via* Akt/Nf-κb pathway. Mol Cell Endocrinol (2016) 437:268–79. doi: 10.1016/j.mce.2016.06.029 27378149

[69] Carneiro MBH Roma EH Ranson AJ Doria NA Debrabant A Sacks DL. Nox2-derived reactive oxygen species control inflammation during leishmania amazonensis infection by mediating infection-induced neutrophil apoptosis. J Immunol (2018) 200(1):196–208. doi: 10.4049/jimmunol.1700899 29158417

[70] Yoshida H Okabe Y Kawane K Fukuyama H Nagata S. Lethal anemia caused by interferon-beta produced in mouse embryos carrying undigested DNA. Nat Immunol (2005) 6(1):49–56. doi: 10.1038/ni1146 15568025

[71] Wang J Li X Bello BK Yu G Yang Q Yang H. Activation of Tlr2 heterodimers-mediated nf-κb, mapk, akt signaling pathways is responsible for *vibrio alginolyticus* triggered inflammatory response *in vitro* . Microbial Pathogenesis (2022) 162:105219. doi: 10.1016/j.micpath.2021.105219 34601054

[72] Dhanasekaran DN Reddy EP. Jnk signaling in apoptosis. Oncogene (2008) 27(48):6245–51. doi: 10.1038/onc.2008.301 PMC3063296 18931691

[73] Krajarng A Imoto M Tashiro E Fujimaki T Shinjo S Watanapokasin R. Apoptosis induction associated with the er stress response through up-regulation of jnk in hela cells by gambogic acid. BMC Complementary Altern Med (2015) 15:26. doi: 10.1186/s12906-015-0544-4 PMC4340837 25887496

[74] Wagner EF Nebreda AR. Signal integration by jnk and P38 mapk pathways in cancer development. Nat Rev Cancer (2009) 9(8):537–49. doi: 10.1038/nrc2694 19629069

[75] Lee C Kim Y Jeon JH. Jnk and P38 mitogen-activated protein kinase pathways contribute to porcine epidemic diarrhea virus infection. Virus Res (2016) 222:1–12. doi: 10.1016/j.virusres.2016.05.018 27215486 PMC7114560

[76] Kumar A Singh UK Kini SG Garg V Agrawal S Tomar PK. Jnk pathway signaling: A novel and smarter therapeutic targets for various biological diseases. Future Med Chem (2015) 7(15):2065–86. doi: 10.4155/fmc.15.132 26505831

[77] Islam MT Ali ES Uddin SJ Islam MA Shaw S Khan IN. Andrographolide, a diterpene lactone from andrographis paniculata and its therapeutic promises in cancer. Cancer Lett (2018) 420:129–45. doi: 10.1016/j.canlet.2018.01.074 29408515

[78] Yan GR Zhou HH Wang Y Zhong Y Tan ZL Wang Y. Protective effects of andrographolide analogue Al-1 on ros-induced rin-mβ cell death by inducing ros generation. PloS One (2013) 8(6):e63656. doi: 10.1371/journal.pone.0063656 23750203 PMC3672203

[79] Yen TL Chen RJ Jayakumar T Lu WJ Hsieh CY Hsu MJ. Andrographolide stimulates P38 mitogen-activated protein kinase-nuclear factor erythroid-2-Related factor 2-heme oxygenase 1 signaling in primary cerebral endothelial cells for definite protection against ischemic stroke in rats. Trans Res J Lab Clin Med (2016) 170:57–72. doi: 10.1016/j.trsl.2015.12.002 26746802

[80] Wang S Li H Chen S Wang Z Yao Y Chen T. Andrographolide induces apoptosis in human osteosarcoma cells *Via* the Ros/Jnk pathway. Int J Oncol (2020) 56(6):1417–28. doi: 10.3892/ijo.2020.5032 PMC7170044 32236589

