# Supplementary material for: Andrographolide promote the growth and immunity of Litopenaeus vannamei, and protects shrimps against Vibrio alginolyticus by regulating inflammation and apoptosis via a ROS-JNK dependent pathway
Source: Front Immunol. 2022 Sep 9;13:990297. doi: 10.3389/fimmu.2022.990297 (PMC9505992; doi:10.3389/fimmu.2022.990297)
Supplement: Supplementary file 2 [file Table_1.docx]

**Table S1.** Sequences of primers used in this study

| **Name** | **Sequence (5′-3′)** |
| --- | --- |
| **For RT-qPCR** |  |
| RT-LvLSZ-F | TTCCGATCTGATGTCCGATGA |
| RT-LvLSZ-R | TTGCTGTTGTAAGCCACCCAG |
| RT-LvPO-F | GAACTCCATTCCGTCCGTCTG |
| RT-LvPO-R | CGGCTTCGCTCTGGTTAGG |
| RT-LvSOD-F | GCGTTGGAGTGAAAGGCTCT |
| RT-LvSOD-R | TCACGTAATCTGCACGGAGG |
| RT-LvCrustin-F | ATTCTGTGCGGCCTCTTTAC |
| RT-LvCrustin-R | ATCGGTCGTTCTTCAGATGG |
| RT-LvPEN3-F | GGGTTTCCTCCTGCGTCCG |
| RT-Lv PEN3-R | GAAGGGTGGTGGCCTGGGT |
| RT-LvALF1-F | ACAGGCTTCCGAGCAACAC |
| RT-LvALF1-R | TGGCACAAGAGCAATCAGG |
| RT-LvCAT-F | GAGGCCGTCTACTGCAAGTT |
| RT-LvCAT-R | GAGGGGTAATCGCCACTTGA |
| RT-LvGPX-F | AGGGACTTCCACCAGATG |
| RT-LvGPX-R | CAACAACTCCCCTTCGGTA |
| RT-LvGST-F | AAGATAACGCAGAGCAAGG |
| RT-LvGST-R | TCGTAGGTGACGGTAAAGA |
| RT-LvP53-F | CGAATCCCCACATCCACG |
| RT-LvP53-R | GGCGGCTGATACACCACC |
| RT-LvBax-F | GGTGGAATCACAAGAGAGCGA |
| RT-LvBax-R | TGTTCTCCACGGTGTCTCAC |
| RT-LvBcl-2-F | CCTTGCTTGACACAGTCGGA |
| RT-LvBcl-2-R | CAGACAAGGTCGTGAGGTGG |
| RT-LvCaspase3-F | AGTTAGTACAAACAGATTGGAGCG |
| RT-LvCaspase3-R | TTGTGGACAGACAGTATGAGGC |
| RT-LvIL-1β-F | CATCCCATTTGTGGTTCTG |
| RT-LvIL-1β-R | TCGTGCTTCACTATGCCTC |
| RT-LvTNFα-F | TCAGCCATCTCCTTCTTGCC |
| RT-LvTNFα-R | CTCCTCCCATCTTCCTTCCC |
| RT-LvJNK-F | GTGCCAGGTGATACAAAT |
| RT-LvJNK-R | CCGTGCTAAACCAAAGTC |
| RT-LvIntegrin-F | CTCAATAACAAGGGCGAGTA |
| RT-LvIntegrin-R | ACGGGAATAAACTGACGACT |
| RT-LvMas-like-F | CAGTCGTTTGACCGCATTT |
| RT-LvMas-like-R | GCGATGATTCCAAGCCTCT |
| RT-LvPeroxinectin-F | AACCTGGCTTGACTGCTATT |
| RT-LvPeroxinectin-R | CTGCCACCACAAACCTTCTA |
| RT-LvDynamin-F | TGGTACTAAGTCCCGTGTTGTCT |
| RT-LvDynamin-R | ATTCCTCCGAGCTGGTGTAT |
| RT-Lvβ-actin-F | GCCCATCTACGAGGGATA |
| RT-Lvβ-actin-R | GGTGGTCGTGAAGGTGTAA |
